# Amoxicillin-non-susceptible *Streptococcus pneumoniae* causing invasive pneumonia: serotypes, clones, and clinical impact

**DOI:** 10.1128/aac.00237-25

**Published:** 2025-07-08

**Authors:** Jordi Càmara, Inmaculada Grau, Aida González-Díaz, Naiara Santos, Fe Tubau-Quintano, Sara Martí, Judith Peñafiel, María Ángeles Domínguez, Josefina Liñares, Román Pallarés, Carmen Ardanuy

**Affiliations:** 1 Department of Clinical Microbiology, Hospital Universitari de Bellvitge-IDIBELL 115336 , L'Hospitalet de Llobregat, Spain; 2 Research Network for Respiratory Diseases (CIBERES-CB06/06/0037), ISCIII 38176 , Madrid, Spain; 3 Infectious Diseases Department, Hospital Universitari de Bellvitge-IDIBELL 115336 , L'Hospitalet de Llobregat, Spain; 4 Biostatistics Unit, Bellvitge Biomedical Research Institute-IDIBELL 115336 , L'Hospitalet de Llobregat, Spain; 5 Department of Medicine, University of Barcelona 368901, Barcelona, Spain; 6 Department of Pathology and Experimental Therapeutics, University of Barcelona 16724, Barcelona, Spain; 7 Research Network for Infectious Diseases (CIBERINFEC-CB21/13/00009), ISCIII 38176 , Madrid, Spain; 8 Department of Clinical Sciences, University of Barcelona 16724, Barcelona, Spain; Houston Methodist Hospital and Weill Cornell Medical College, Houston, Texas, USA

**Keywords:** *Streptococcus pneumoniae*, pneumonia, amoxicillin, mortality, invasive pneumococcal disease

## Abstract

Amoxicillin is one of the most commonly used antibiotics to treat pneumococcal infections. We analyzed the epidemiology and clinical impact of amoxicillin non-susceptibility in invasive pneumococcal pneumonia. This is an observational study based on prospectively collected data at Bellvitge University Hospital (Barcelona, Spain) from 1994 to 2020. Isolates were tested for antibiotic susceptibility, serotyped, and genotyped. Clinical characteristics and 30 day mortality were evaluated using independent Cox proportional hazards models. We analyzed 1,663 episodes. While the proportion of isolates susceptible to amoxicillin increased from 72.9% (1994–2001) to 91.4% (2016–2020), the proportion of isolates with MIC > 2 mg/L remained stable (4.8% and 5.3%, respectively). A single lineage (GPSC6; serotypes 9V, 14, and 11A) accounted for 56.5% of non-susceptible strains. High McCabe scores (ultimately fatal: HR 2.87 [2.10–3.92] and rapidly fatal: HR 4.98 [3.50–7.09]), along with parameters linked to disease severity such as leukopenia (HR 2.14 [1.63–2.82]), shock (HR 3.47 [2.63–4.57]), respiratory failure (HR 4.26 [2.63–6.89]), and bilobar pneumonia (HR 1.44 [1.09–1.90]), were associated with 30 day mortality. The risk of mortality was higher in episodes treated with amoxicillin than with third-generation cephalosporins (HR 1.81 [1.28–2.56]), especially in episodes with amoxicillin MIC > 2 mg/L (HR 6.14 [1.65–22.80]). Amoxicillin therapy was not associated with increased mortality in patients with a non-fatal McCabe score (HR 1.49 [0.62–3.55]), but it was in the ultimately fatal/rapidly fatal group (HR 1.97 [1.34–2.90]). In addition to the crucial importance of host factors in the outcome of invasive pneumococcal pneumonia, our data indicate that amoxicillin yields poorer outcomes compared to third-generation cephalosporins, particularly in patients with a worse prognosis.

## INTRODUCTION

Respiratory tract infections are an important cause of mortality worldwide, with *Streptococcus pneumoniae* being one of their main etiologies (1). Among the currently available antibiotics to treat pneumococcal infections, amoxicillin, alone or in combination, remains a recommended first-line option (2). Because of its favorable properties, i.e., oral administration, high bioavailability, and good pulmonary distribution, amoxicillin is also the preferred choice for non-severe infections. Therefore, the study of the molecular basis and the clinical impact of the non-susceptibility to amoxicillin in *S. pneumoniae* is of special interest.

Due to its genetic plasticity, the pneumococcus is capable of evolving rapidly in response to environmental changes such as antibiotic pressure or conjugate vaccines (3). In this way, increased MIC to β-lactam antibiotics in *S. pneumoniae* is associated with amino acid changes in penicillin-binding proteins (PBPs) (4), acquired through horizontal DNA transfer from commensal streptococci that act as reservoirs of resistance (5). This plasticity is necessary not only to acquire antimicrobial resistance but also to allow compensatory mutations to restore the fitness loss, being a mechanism through which the pneumococcus stabilizes the resistance genotypes (6). The location of these changes determines the impact on the different β-lactam antibiotics. It is generally assumed that changes in PBP2B or PBP2X cause low levels of penicillin resistance, and additional substitutions in PBP1A are required for high-level resistance (7, 8). Among the global population of pneumococci, a few clones associated with invasive disease have successfully incorporated and maintained genetic modifications associated with resistance over time (9). In previous works, we have extensively studied the genetic evolution and the European expansion of PMEN3 (GPSC6), a β-lactam-resistant clone prevalent in Spain that, after several recombination events, currently displays a higher amoxicillin MIC than that of penicillin (10, 11).

While the impact of penicillin and cefotaxime MICs on the outcome of pneumococcal infections has been established for years (12, 13), the level of amoxicillin non-susceptibility leading to poorer outcomes is less clear. In fact, there are important differences in the current amoxicillin MIC breakpoints for non-meningitis indications (oral administration) between the CLSI (resistance ≥ 8 mg/L) and EUCAST (resistance > 1 mg/L) organizations (14, 15). These breakpoints have important implications from a public health perspective as they determine the proportion of resistant strains and thus the feasibility of using amoxicillin as empirical therapy. Therefore, the analysis of the potential impact of elevated MICs in an antibiotic as widely used as both empirical and targeted therapy could be extremely useful. In this study, we analyzed the epidemiology, molecular basis, and clinical impact of amoxicillin non-susceptibility in invasive pneumococcal pneumonia. In particular, we aimed to explore the potential role of amoxicillin, both for empirical treatment and antibiotic de-escalation, in the context of invasive pneumonia.

## MATERIALS AND METHODS

### Study setting and clinical data

This was an observational study based on prospectively collected data. Hospital Universitari de Bellvitge (HUB) is a 700 bed teaching hospital that admits adult patients (≥18 years old) in the Barcelona Metropolitan area. Over the past decades, all invasive pneumococcal disease (IPD) episodes detected at HUB were prospectively collected and recorded in a database. Recorded data includes age, sex, origin of infection, acquisition (extrahospitalary or nosocomial), comorbidities, severity of underlying diseases (McCabe and Jackson score), treatment, and 30 day mortality. IPD definition is restricted to the isolation of *S. pneumoniae* from a normally sterile body site (https://ndc.services.cdc.gov/case-definitions/invasive-pneumococcal-disease-2017). For this study, we focused on invasive pneumococcal pneumonia episodes (radiologically confirmed) in the 1994–2020 period. To avoid overrepresentation of patients with multiple episodes, only each patient’s first episode was considered for mortality analysis. Three treatment groups were defined: amoxicillin (amoxicillin/amoxicillin-clavulanate), third-generation cephalosporines (3GC; ceftriaxone/cefotaxime), and others (antibiotics not included in the previous groups or antibiotic combinations). Only patients who received at least 48 hours of a particular antibiotic were included.

### Bacterial isolates, antimicrobial susceptibility, and molecular typing

*S. pneumoniae* isolates were identified by conventional procedures (optochin susceptibility and/or bile solubility) and serotyped at the Spanish Pneumococcal Reference Laboratory for Pneumococci (Quellung, dot blot, and/or capsular sequence typing). Antimicrobial susceptibility was tested by microdilution following the CLSI recommendations. According to the amoxicillin MIC, isolates were divided into three groups: ≤0.5, >0.5–2, and >2 mg/L. Molecular typing with PFGE (*SmaI*) was performed on all available isolates. A selection belonging to major clusters (>10 isolates) was studied by MLST (https://pubmlst.org/). To characterize the molecular basis of amoxicillin resistance, 54 randomly selected isolates from the five most prevalent lineages were analyzed using whole-genome sequencing (WGS).

### Whole-genome sequencing

WGS was performed as previously described (16). DNA was extracted (QIAamp DNA Mini Kit, Qiagen), quantified (Qubit, Thermo Fisher Scientific), and sequenced using Nextera XT (paired-end sequencing, 2 × 300 bp) in a MiSeq platform (Illumina). Sequences were assembled using the INNUca v4.2 pipeline (github.com/B-UMMI/INNUca). A whole genome alignment created by Snippy (github.com/tseemann/snippy) was used to construct a phylogenetic tree with Gubbins (17). Amino acid changes in proteins related to β-lactam resistance (PBP1A, PBP2B, PBP2X, MurM, and MurN) were analyzed with Geneious R9, using R6 (NC_003098) as reference. PBP alleles were determined using Pathogenwatch (pathogen.watch/), which uses the CDC *Streptococcus* Laboratory scheme (https://www.cdc.gov/strep-lab/php/pneumococcus/mics.html). The phylogenetic tree and associated metadata representation were visualized using iTOL (itol.embl.de/). Reads were deposited in the European Nucleotide Archive, and metadata are summarized in Table S1.

### Statistical analysis

To compare the clinical profile by study group, the Analysis of Variance was used for continuous variables and the Pearson’s Chi-squared test or Fisher’s exact test for categorical variables. Mortality was represented by a Kaplan-Meier plot and compared using a log-rank test. Multivariate Cox proportional hazards models were used to examine factors associated with mortality for each MIC group. All variables were checked for noncollinearity (variance inflation factor < 5). The McCabe score was included in model 1, and it was replaced by individual comorbidities in model 2. Models were repeated, excluding episodes that resulted in death within the first 24 hours. Hazard ratios (HRs) are reported with 95% confidence intervals. All analyses were done with the statistical package R version 4.3.2 for Windows.

## RESULTS

### Epidemiology and evolution of amoxicillin non-susceptibility

From 1994 to 2020, we collected 1,663 *S*. *pneumoniae* isolates. Serotype was available for 1,629 of them (98.0%), with seven serotypes accounting for half of the episodes: 3 (*n* = 203, 12.5%), 14 (*n* = 119, 7.3%), 19A (*n* = 113, 6.9%), 1 (*n* = 112, 6.9%), 8 (*n* = 92, 5.6%), 7F (*n* = 91, 5.6%), and 9V (*n* = 82, 5.0%). The most recent episodes (2016–2020) were dominated by non-PCV13 serotypes (173/238, 72.7%), with serotypes 8 (*n* = 36, 15.1%), 3 (*n* = 27, 11.3%), and 12F (*n* = 15, 6.3%) being the most frequent.

**Fig 1:**
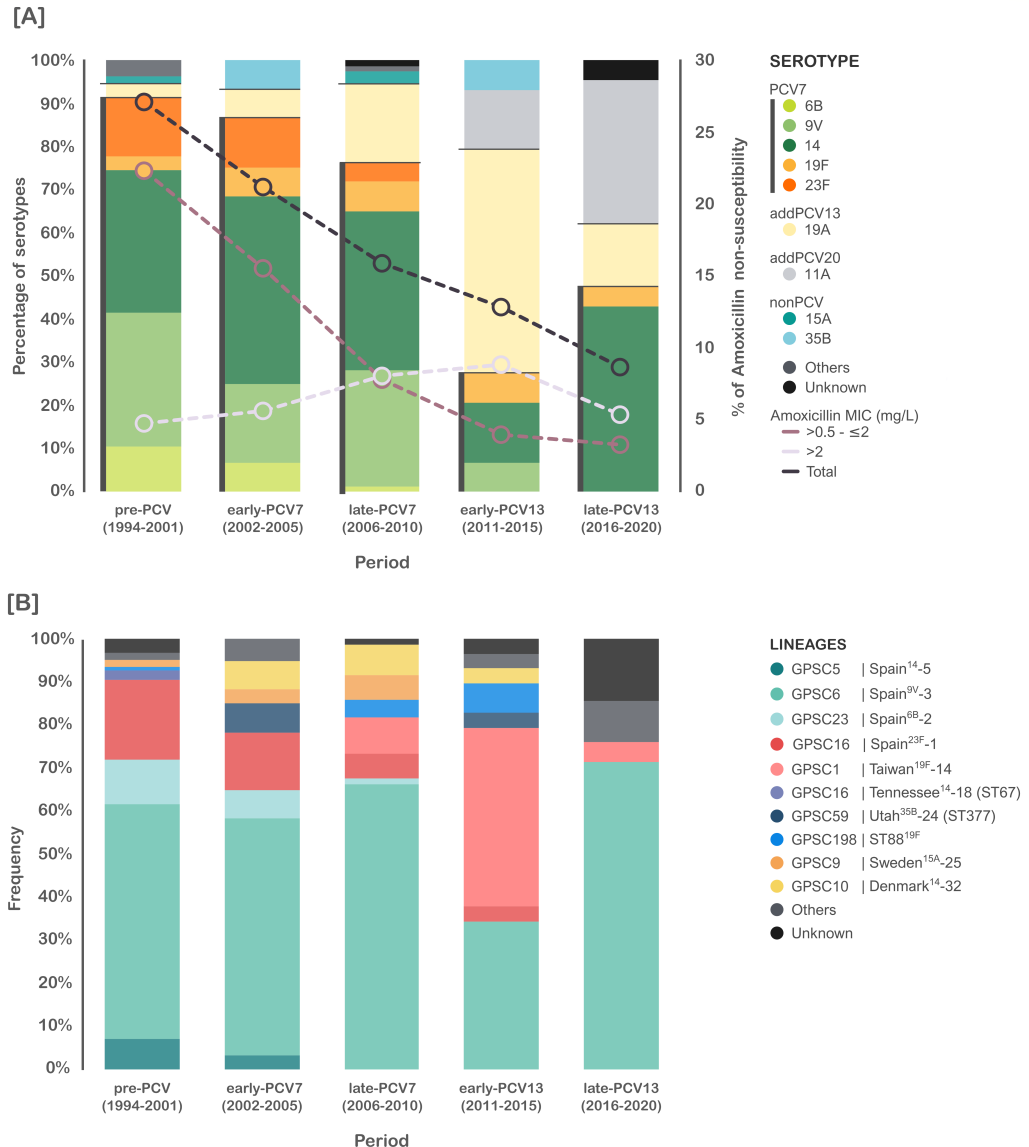
Evolution of amoxicillin susceptibility, pneumococcal serotypes (**A**), and genetic lineages (**B**) associated with amoxicillin non-susceptibility. Serotypes are classified according to the pneumococcal conjugate vaccines PCV7, PCV13, and PCV20. Periods were defined according to the introduction of PCVs for children in Spain (PCV7 in 2001 and PCV13 in 2010). Lineages correspond to the Global Pneumococcal Sequence Clusters (GPSC) and Pneumococcal Molecular Epidemiology Network (PMEN) clones. Serotypes or lineages representing less than three isolates were grouped into “others.” “Unknown” refers to isolates not available for typing.

The proportion of isolates susceptible to amoxicillin increased over time from 72.9% (1994–2001) to 91.4% (2016–2020). This was associated with a drastic reduction of isolates with amoxicillin MICs > 0.5–2 mg/L, whose proportion fell from 22.3% to 3.3% (Fig. 1A). In contrast, the proportion of isolates with amoxicillin MIC > 2 mg/L remained stable (4.8% and 5.3%). Amoxicillin non-susceptible isolates corresponded mostly to vaccine serotypes 14, 9V (lineage GPSC6, Spain^9V^-3), and 23F (lineage GPSC16, Spain^23F^-1) before the introduction of PCVs (Fig. 1B). Serotypes 19A (GPSC1, Taiwan^19F^-14) and 11A (GPSC6) emerged as predominant causes of amoxicillin resistance after the introduction of PCV7 and PCV13, respectively. The GPSC6 lineage, which currently expresses serotype 11A (formerly serotypes 9V and 14), has remained the primary contributor to amoxicillin non-susceptibility over time.

**Fig 2:**
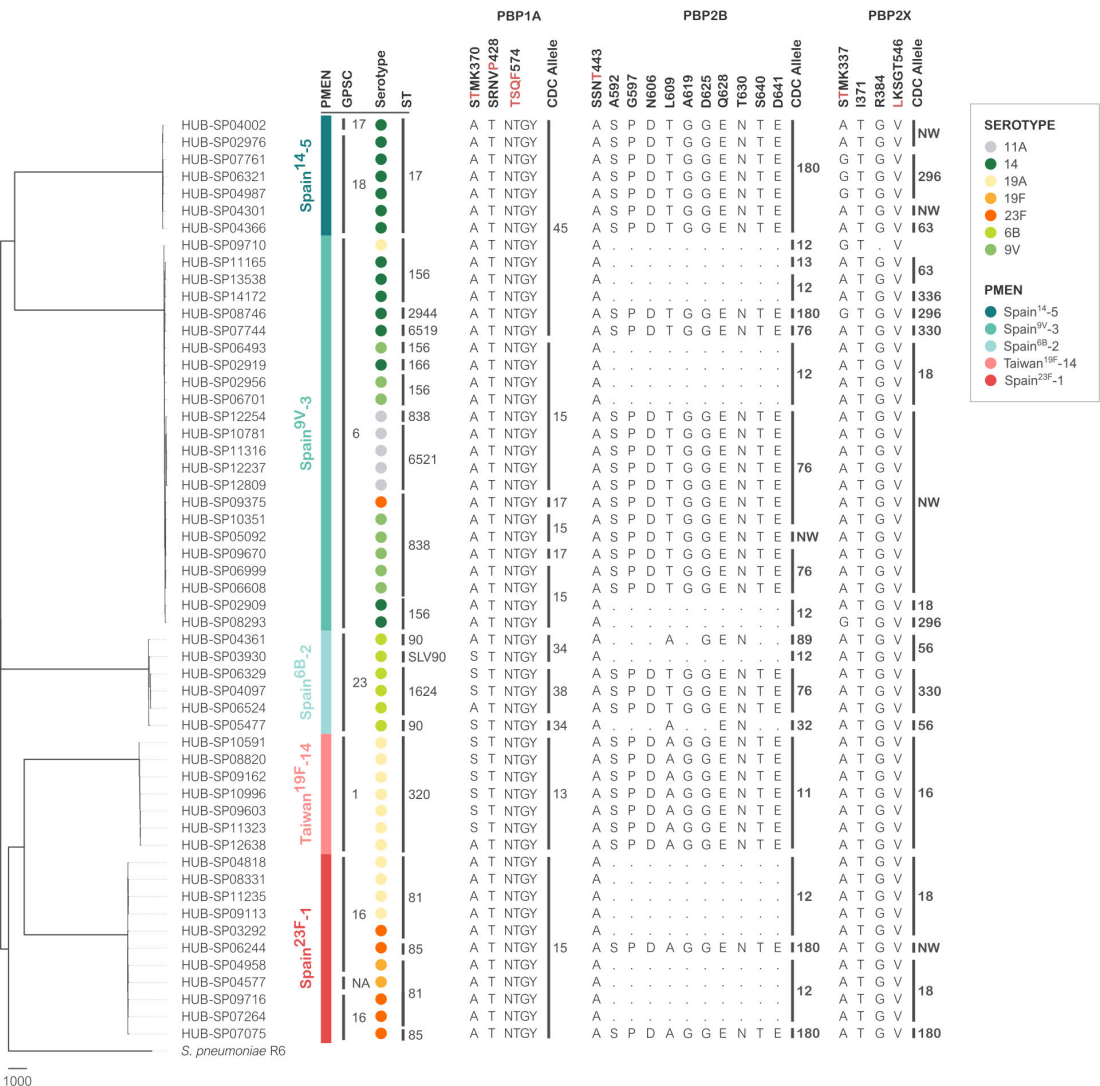
Whole-genome sequencing analysis of major clones associated with amoxicillin non-susceptibility. The figure shows the lineages (GPSC and PMEN clones), the serotypes, and the amino acid changes in PBP1A, PBP2B, and PBP2X proteins associated with β-lactam resistance. Changes in protein motifs are highlighted in red. GPSC, Global Pneumococcal Sequencing Cluster; PMEN, Pneumococcal Molecular Epidemiology Network; and ST, sequence type.

A selection of isolates corresponding to the five most frequent amoxicillin-non-susceptible lineages (GPSC6/Spain^9V^-3, GPSC1/Taiwan^19F^-14, GPSC16/Spain^23F^-1, GPSC5/Spain^14^-5, and GPSC23/Spain^6B^-2) was studied through WGS (Fig. 2; Table S1). As described previously, all amoxicillin-non-susceptible isolates had characteristic changes in PBP1A, PBP2X, PBP2B, and MurM proteins (Tables S2A through E) (8). In particular, the P432T change and the alteration of 574TSQF to 574NTGY (PBP1A), the T446A in the 443SSNT motif (PBP2B), the I371T and L546V changes (PBP2X), and the V101A substitution (MurM) were present in all isolates, irrespective of their genetic background. Regarding isolates with high amoxicillin MIC (>2 mg/L), all showed alterations in the 590–641 region of PBP2B (18, 19).

### Impact of amoxicillin MIC on clinical outcome

**Fig 3:**
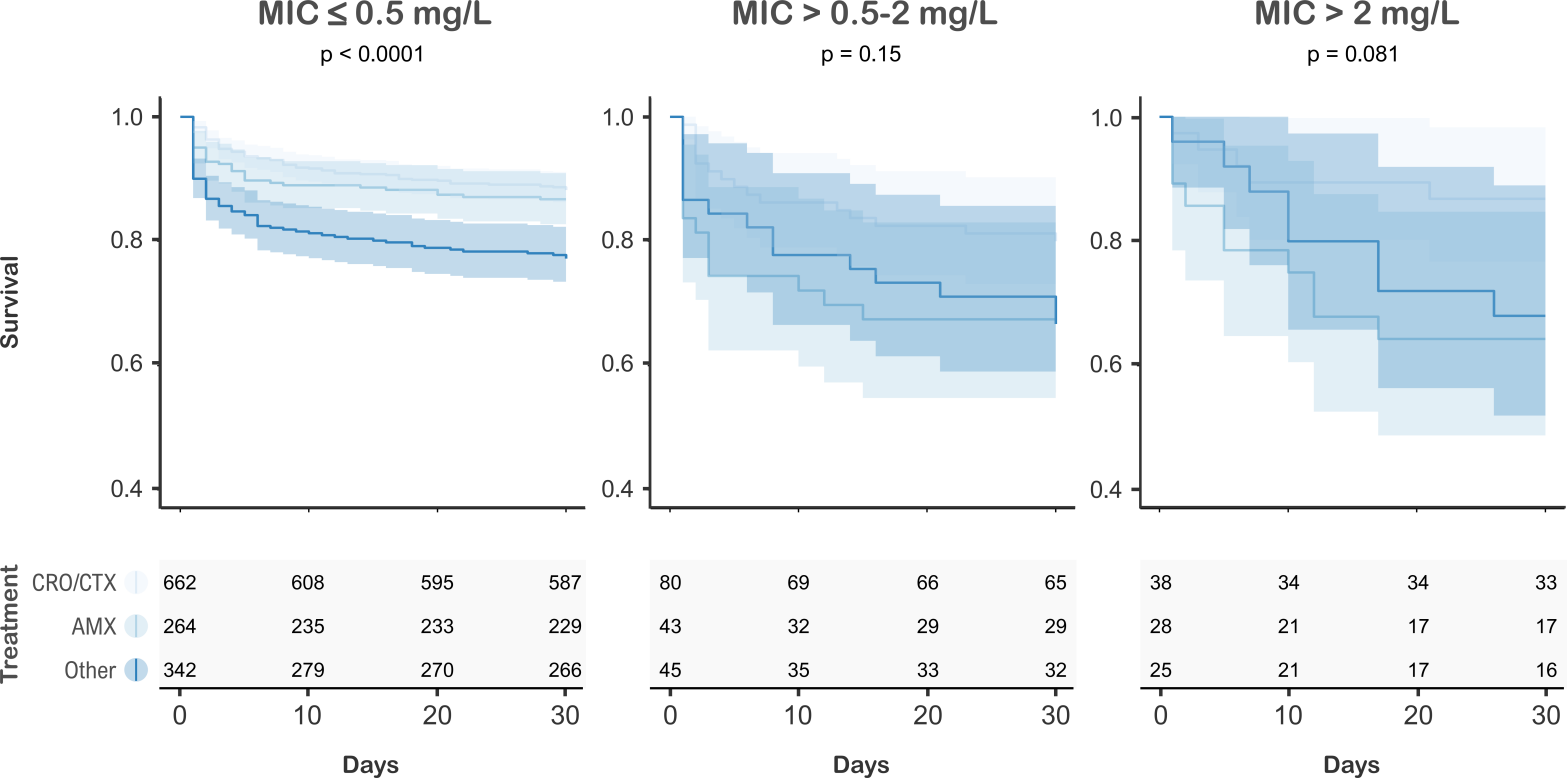
Kaplan-Meier survival curves. Episodes were classified according to the amoxicillin MIC and the antimicrobial administered. The figure shows confidence intervals and *P*-values (log-rank test). “Other” includes episodes in which antibiotics other than amoxicillin and third-generation cephalosporins were administered. AMX, amoxicillin; CRO/CTX, ceftriaxone/cefotaxime.

Of 1,592 first episodes, 52 were excluded due to polymicrobial infection, and 13 were excluded because patients did not receive antimicrobial treatment or data were missing. Therefore, 1,527 episodes were considered for statistical analysis. The median age was 62.4 years (SD 17.9), and 36.7% were women (Table 1). Amoxicillin-resistant isolates occurred in older patients (68.9 years, SD 17.3) and with worse prognosis (58.3% ultimately/rapidly fatal McCabe score). The proportion of patients with malignancies (38.5%) and under immunosuppressive therapy (29.7%) was also higher in this group. Compared with amoxicillin, episodes treated with 3GC more frequently presented parameters associated with the severity of the disease such as leukopenia (14.0% vs 17.6%), respiratory failure (46.0% vs 60.9%), shock (11.3% vs 15.5%), and bilobar pneumonia (24.3% vs 33.2%). The group of amoxicillin therapy displayed higher rates of nosocomial acquisition (8.7% vs 2.9%) and rapidly fatal McCabe score (14.6% vs 8.7%). Regarding patients who received other treatments, it should be noted that they represent a heterogeneous group with more complex diseases and greater severity of infection. Consequently, this group includes an overrepresentation of broad-spectrum treatments due to the patients’ own characteristics, which may indicate a prescription bias. Crude mortality showed a similar trend for amoxicillin and 3GC in episodes caused by amoxicillin-susceptible isolates (Fig. 3). Among episodes caused by isolates with MIC > 0.5–2 or >2 mg/L, amoxicillin therapy was associated with a statistically nonsignificant increased crude mortality.

**TABLE 1:** Clinical characteristics of the study population by antimicrobial treatment and amoxicillin MIC^*a*^

Clinical characteristics	Antimicrobial treatment				Amoxicillin MIC (mg/L)				All episodes (*n* = 1,527)
	AMX (*n* = 335)	CRO/CTX (*n* = 780)	Other (*n* = 412)	*P*-value	≤0.5 (*n* = 1,268)	>0.5–2 (*n* = 168)	>2 (*n* = 91)	*P*-value	
Age mean (SD)	62.2 (19.6)	62.7 (18.0)	62.0 (16.3)	0.742	62.0 (17.7)	61.8 (19.0)	68.9 (17.3)	**0.002**	62.4 (17.9)
Female sex	117 (34.9%)	291 (37.3%)	153 (37.1%)	0.737	462 (36.4%)	58 (34.5%)	41 (45.1%)	0.211	561 (36.7%)
Underlying conditions									
Alcohol abuse	48 (14.8%)	100 (13.0%)	67 (16.6%)	0.241	179 (14.4%)	29 (18.0%)	7 (7.7%)	0.081	215 (14.4%)
Current smoking	120 (36.9%)	270 (35.2%)	155 (38.4%)	0.542	459 (36.9%)	68 (42.0%)	18 (19.8%)	**0.001**	545 (36.4%)
Acquisition				**<0.001**				**0.008**	
Nosocomial	29 (8.7%)	23 (2.9%)	34 (8.3%)		62 (4.9%)	18 (10.7%)	6 (6.6%)		86 (5.6%)
Extrahospitalary	306 (91.3%)	756 (97.0%)	378 (91.7%)		1,205 (95.1%)	150 (89.3%)	85 (93.4%)		1,440 (94.4%)
Comorbidities									
Diabetes mellitus	78 (23.3%)	166 (21.3%)	81 (19.7%)	0.485	271 (21.4%)	39 (23.2%)	15 (16.5%)	0.442	325 (21.3%)
Malignancies	88 (26.3%)	187 (24.0%)	116 (28.2%)	0.276	315 (24.8%)	41 (24.4%)	35 (38.5%)	**0.015**	391 (25.6%)
Liver cirrhosis	22 (6.6%)	63 (8.1%)	37 (9.0%)	0.477	100 (7.9%)	16 (9.5%)	6 (6.6%)	0.671	122 (8.0%)
Chronic renal failure (E IV-V)	9 (2.69%)	27 (3.46%)	13 (3.2%)	0.795	40 (3.2%)	6 (3.6%)	3 (3.3%)	0.874	49 (3.2%)
Chronic pulmonary disease	70 (20.9%)	178 (22.8%)	107 (26.0%)	0.243	310 (24.4%)	30 (17.9%)	15 (16.5%)	**0.048**	355 (23.2%)
HIV infection	30 (9.0%)	91 (11.7%)	24 (5.8%)	**0.004**	109 (8.6%)	30 (17.9%)	6 (6.6%)	**<0.001**	145 (9.5%)
Chronic heart disease	60 (17.9%)	168 (21.5%)	72 (17.5%)	0.162	245 (19.3%)	30 (17.9%)	25 (27.5%)	0.138	300 (19.6%)
Cerebrovascular disease/dementia	41 (12.2%)	48 (6.2%)	20 (4.9%)	**<0.001**	88 (6.9%)	10 (6.0%)	11 (12.1%)	0.150	109 (7.1%)
Immunosuppressive therapy	62 (18.5%)	142 (18.2%)	89 (21.6%)	0.344	229 (18.1%)	37 (22.0%)	27 (29.7%)	**0.015**	293 (19.2%)
Clinical presentation									
Neutropenia	7 (2.1%)	20 (2.6%)	40 (9.7%)	**<0.001**	57 (4.5%)	6 (3.6%)	4 (4.4%)	0.905	67 (4.4%)
Leukopenia	47 (14.0%)	137 (17.6%)	96 (23.3%)	**0.004**	233 (18.4%)	38 (22.6%)	9 (9.9%)	**0.041**	280 (18.3%)
Shock at presentation	38 (11.3%)	121 (15.5%)	117 (28.4%)	**<0.001**	227 (17.9%)	34 (20.2%)	15 (16.5%)	0.700	276 (18.1%)
Respiratory failure	150 (46.0%)	467 (60.9%)	249 (62.1%)	**<0.001**	723 (58.3%)	89 (54.6%)	54 (60.0%)	0.621	866 (58.0%)
Bilobar pneumonia	81 (24.3%)	257 (33.2%)	157 (38.5%)	**<0.001**	418 (33.2%)	52 (31.1%)	25 (27.8%)	0.513	495 (32.7%)
Pleural effusion	78 (23.3%)	191 (24.6%)	86 (21.0%)	0.365	298 (23.6%)	38 (22.6%)	19 (20.9%)	0.812	355 (23.4%)
Bacteriemia	305 (91.0%)	743 (95.3%)	391 (94.9%)	**0.017**	1,200 (94.6%)	156 (92.9%)	83 (91.2%)	0.287	1,439 (94.2%)
McCabe and Jackson score				**0.022**				**<0.001**	
Non-fatal	180 (53.7%)	487 (62.4%)	244 (59.2%)		788 (62.1%)	85 (50.6%)	38 (41.8%)		911 (59.7%)
Ultimately fatal	106 (31.6%)	225 (28.8%)	124 (30.1%)		352 (27.8%)	63 (37.5%)	40 (44.0%)		455 (29.8%)
Rapidly fatal	49 (14.6%)	68 (8.7%)	44 (10.7%)		128 (10.1%)	20 (11.9%)	13 (14.3%)		161 (10.5%)
30 day mortality	59 (17.6%)	100 (12.8%)	101 (24.5%)	**<0.001**	192 (15.1%)	45 (26.8%)	23 (25.3%)	**<0.001**	260 (17.0%)

^*a*^ Data are presented as the number of episodes and the percentage among available data, otherwise stated. Statistically significant values (*P* < 0.05) are highlighted in bold. AMX, amoxicillin and CRO/CTX, ceftriaxone/cefotaxime.

**Fig 4:**
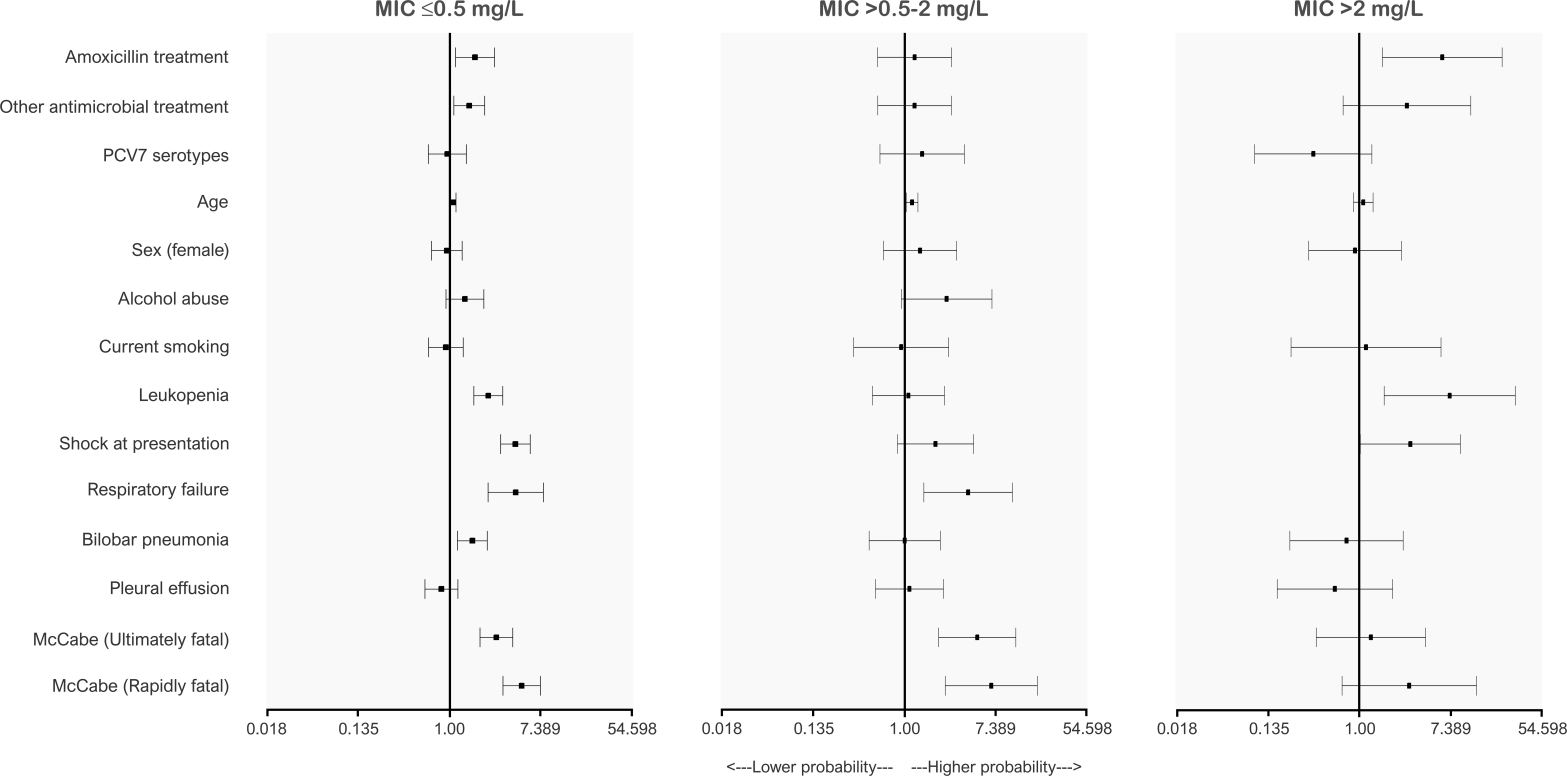
Multivariate analysis of factors associated with 30 day mortality by amoxicillin MIC. Third-generation cephalosporins (ceftriaxone/cefotaxime) were set as reference treatment. “Other antimicrobial treatment” refers to antimicrobials other than amoxicillin and third-generation cephalosporins. Age was included in the model as 5 year period increments. McCabe “Ultimately fatal” and “Rapidly fatal” estimates are referenced to the McCabe “Non-fatal” category. Alcohol abuse and respiratory failure were not included in the MIC > 2 mg/L model due to low numbers. Complete data and additional analysis are included in Tables S3A and B.

The analysis through independent Cox proportional hazards models (Fig. 4; Tables S3A and B) indicated that age, higher McCabe score, leukopenia, shock, respiratory failure, and bilobar pneumonia were consistently associated with 30 day mortality. Overall, amoxicillin therapy exhibited poorer outcomes compared to 3GC in most analyses. Regarding amoxicillin MIC, the estimates remained stable across episodes caused by isolates with MIC ≤ 0.5 mg/L (HR 1.75 [95% CI 1.14–2.68]) and MIC > 0.5–2 mg/L (HR 1.25 [95% CI 0.55–2.80]). However, amoxicillin therapy was clearly associated with increased mortality in episodes involving isolates with MIC > 2 mg/L (HR 6.14 [95% CI 1.65–22.8]). Data were consistent, excluding episodes with mortality within the first 24 hours. Given the significant impact of host factors on mortality, we aimed to analyze differences within each patient group. Thus, we compared episodes with non-fatal and ultimately/rapidly fatal McCabe scores (Table S3C). Notably, amoxicillin therapy was not associated with increased mortality in patients from the non-fatal McCabe group (HR 1.49 [95% CI 0.62–3.55]); however, it was associated with increased mortality in the ultimately or rapidly fatal McCabe group (HR 1.97 [95% CI 1.34–2.90]).

## DISCUSSION

Amoxicillin is one of the most commonly used antibiotics to treat pneumococcal pneumonia. Furthermore, it is also employed in stewardship programs to reduce the antimicrobial spectrum and switch to oral administration (20). However, there are significant differences in the current breakpoints between the EUCAST and CLSI organizations, making this issue controversial. In this work, we show the evolution and the molecular epidemiology of *S. pneumoniae* with increased amoxicillin MICs and its clinical impact on invasive pneumonia.

Despite the extensive use of β-lactam antibiotics over decades, the occurrence of *S. pneumoniae* isolates with high amoxicillin MICs is unusual. Therefore, it is generally accepted that this antibiotic is a safe option to treat pneumococcal infections other than meningitis. In Spain, β-lactam resistance in pneumococci remained high before the introduction of PCVs but has declined in recent years (21). This is largely due to a reduction in vaccine lineages associated with β-lactam resistance, such as GPSC16/Spain^23F^-1, GPSC6/Spain^9V^-3, and GPSC1/Taiwan^19F^-14 (22). In our study, we observed a clear reduction in the percentage of amoxicillin non-susceptibility from 27% in the pre-PCVs period to the current 9%. This highlights the enormous impact of the introduction of PCVs on pneumococcal epidemiology and the benefits in terms of reducing antimicrobial resistance (23). However, the rate of isolates with high amoxicillin MICs remained stable because of the persistence of the GPSC6/Spain^9V^-3 lineage, currently expressing serotype 11A, which is not included in PCV13 (16). As expected, lineages showing amoxicillin non-susceptibility had a similar pattern of changes in PBP1A, 2B, and 2X, as well as substitutions in MurM. This indicates not only its acquisition from horizontal gene transfer but also the success of this particular pattern, either in expressing the resistance phenotype or compensating for resistance mutations (6, 8).

For β-lactam antibiotics, the PK/PD parameter that best correlates with efficacy is the duration of the dosing interval for which the antibiotic concentration is above the MIC, with 40% being the target duration (24). Regarding amoxicillin, it has been reported that a dosage regimen of 500 mg every 8 hours may be insufficient to achieve the 40%T > MIC in infections caused by strains with MICs over 0.5 mg/L (25). Then, treatment guidelines recommend higher doses, such as 1 g every 8 hours (2). Regarding animal models, a mice model of pneumonia simulating a human dosing regimen of 1 g/8 hours demonstrated that amoxicillin had a bactericidal effect in pneumococcal strains with amoxicillin MICs of 2–4 mg/L (26). However, other models showed worse results after infection with pneumococci with amoxicillin MIC > 1 mg/L (27, 28). Regarding clinical studies, most of the published data support the hypotheses that β-lactam therapy is not associated with impaired outcomes in non-meningeal infections caused by strains with penicillin MIC ≤ 2 mg/L (12, 29–31). However, it is not easy to analyze the impact of antimicrobial resistance on the clinical outcome of pneumococcal infections. On the one hand, some host factors, such as age and comorbidities, are strongly associated with increased mortality (32). On the other hand, there are bacterial factors that can interfere with the analysis. For instance, PCV7 serotypes, which are associated with β-lactam non-susceptibility, have also been described as exhibiting higher virulence (33). Indeed, our data show that host factors, grouped in the McCabe prognostic score, are strongly associated with mortality. Regarding amoxicillin, the results need some consideration. First, amoxicillin-resistant isolates occur in older patients with a worse prognosis. The higher mortality rates observed in this group are consistent with their elevated underlying risk (34). Second, overall results indicate that amoxicillin therapy is associated with a slightly higher risk of 30 day mortality compared to 3GC. This was due to a clearly increased risk in episodes caused by isolates with amoxicillin MIC > 2 mg/L, but we found no evidence of increased risk in episodes caused by isolates with lower MIC. Then, our data align with the current CLSI breakpoints, which categorize isolates with an amoxicillin MIC ≤ 2 mg/L as susceptible. Third, among episodes with MIC > 0.5–2 mg/L, differences in mortality between amoxicillin and 3GC were primarily due to differences in early mortality, that is, mortality that occurred within the first 24 hours. It is traditionally recognized that, in pneumococcal pneumonia, antimicrobial therapy has limited influence on mortality within the first 24 hours (35). However, given that the initial bacterial load is clearly associated with worse outcomes, observed differences in mortality may also partly reflect variations in antimicrobial efficacy within this subgroup of patients (36). Fourth, amoxicillin therapy was independently associated with increased mortality in patients in the McCabe ultimately/rapidly fatal groups, but not in patients in the non-fatal group. These data are interesting as they indicate variations in antimicrobial efficacy based on the host’s status. Taken together, these results highlight that prescribing a specific antimicrobial should always be accompanied by a thorough evaluation of the host’s comorbidities and the severity of the infection.

We consider that this study has several strengths. As far as we know, this is the largest series analyzing the clinical impact of amoxicillin on invasive pneumococcal pneumonia. All episodes had radiologically confirmed pneumococcal pneumonia, data on antimicrobial susceptibility, and molecular epidemiology. Additionally, clinical data were prospectively and systematically collected by the same team of infectious diseases physicians, providing consistency to the results. This study also has limitations that should be considered. Inherent in the retrospective nature of the analysis are unmeasured factors that could have influenced the results. However, the use of multivariate statistical models should enable adjustment for the commonly recognized risk factors. Interactions between risk factors have not been included in the models. Another limitation is the lack of data on pneumococcal vaccination (PPSV23), which could partially affect the outcome of pneumococcal pneumonia. Furthermore, information on amoxicillin dosage was not available. However, the standard recommendation at our institution is 1 g every 8 hours for hospitalized patients, meaning that most patients likely received this dosage. Finally, the results reflect the epidemiology of a specific geographical region with unique host conditions and pneumococcal lineages, which may not be generalizable to other settings.

### Conclusions

In this study, we show an increase in amoxicillin susceptibility in the period following the introduction of PCVs. Nevertheless, the prevalence of isolates with high amoxicillin MICs has remained unchanged due to the persistence of the GPSC6/Spain^9V^-3 lineage. In addition to the crucial importance of host factors in the outcome of invasive pneumococcal pneumonia, our data indicate that amoxicillin treatment yields poorer outcomes compared to 3GC, particularly in patients with a worse prognosis. Therefore, amoxicillin could be a treatment option either for antibiotic de-escalation in stewardship programs or as empirical treatment of pneumococcal pneumonia in stabilized patients with a good prognosis.

## SUPPLEMENTAL MATERIAL

Table S1 aac.00237-25-s0001.xlsx Metadata of amoxicillin non-susceptible *Streptococcus pneumoniae* isolates sequenced through WGS.

Table S2 aac.00237-25-s0002.xlsx Changes in the amino acid sequence of PBP1A, PBP2X, PBP2B, MurM, and MurN proteins.

Table S3 aac.00237-25-s0003.xlsx Analysis of factors associated with 30-day mortality by amoxicillin MIC and McCabe score.

ASM does not own the copyrights to Supplemental Material that may be linked to, or accessed through, an article. The authors have granted ASM a non-exclusive, world-wide license to publish the Supplemental Material files. Please contact the corresponding author directly for reuse.
